# Mucositis Pain and Its Temporal Relationship to White Cell Count

**DOI:** 10.1111/pan.15063

**Published:** 2025-01-06

**Authors:** Claire Douglas, James D. Morse, Brian J. Anderson

**Affiliations:** ^1^ Department of Anaesthesia Starship Children's Hospital Auckland New Zealand; ^2^ Department of Anaesthesiology University of Auckland Auckland New Zealand; ^3^ Department of Pharmacology and Clinical Pharmacology University of Auckland Auckland New Zealand

**Keywords:** acute pain, allometry, mucositis, pharmacodynamics, pharmacokinetics, pharmacometrics

## Abstract

**Background:**

Children who have received chemotherapy and/or radiotherapy treatment resulting in neutropenia can suffer painful mucositis. We explored the relationship between pain score and white cell count in children with mucositis due to immunosuppression and assessed the influence of opioid and ketamine analgesia.

**Methods:**

Children with mucositis nursed in the pediatric oncology and hematology ward were invited to partake in this observational study following referral to the pediatric pain service for intravenous analgesia. Pain scores, white cell count, neutrophil count, and analgesia requirements were recorded daily until intravenous analgesia was either stopped or transitioned to oral analgesia. Data were analyzed using nonlinear mixed effects models that sought a relationship between white cell count and pain score using a sigmoid maximal effect (E_MAX_) model. The impact of analgesic use on pain score was determined. The temporal relationship between white cell count and pain score was characterized by using a delayed effect model with an equilibration half‐time.

**Results:**

Fifty children were enrolled in the study from January 2022 to December 2023. The equilibration half‐time relating the rise in white cell count and pain response was 0.29 days. The initial pain score (estimated in those children already started on treatment with paracetamol and tramadol) was 6.3 (maximum pain 10). The maximum pain reduction was 59% of that initial pain score. Morphine and ketamine further reduced pain; the maximum response for opioids was 38% reduction and that for ketamine was 11%.

**Conclusion:**

Pain relief from mucositis is related to an increase in white cell count after a period of severe neutropenia, where white cell count is a surrogate for neutrophil count. There is a delay in analgesic response of approximately 1 day. This analgesic response to increasing white cell count had greater dominance than analgesia achieved using either opioids or ketamine.

## 1 Introduction

Mucositis involving the gastrointestinal tract is a common, painful, and debilitating condition affecting children following chemotherapy and/or radiation for oncological diseases and hematopoietic stem cell transplant. This inflammatory process causes pain and difficulties in functions like eating and swallowing, resulting in lower quality of life and greater need of treatment with opioids and parenteral nutrition [1]. Mucositis onset and resolution has been associated with neutrophil count [2]. The severity of oral mucositis and number of pain medications administered increases as neutrophil count decreases. Neutrophil count recovery coincides with resolution of oral mucositis [3].

Although a number of interventions have been demonstrated to prevent or reduce the severity of mucositis, (e.g., aloe vera, amifostine, cryotherapy, granulocyte‐colony stimulating factor (G‐CSF), intravenous glutamine, honey, keratinocyte growth factor, laser, olymixin/tobramycin/amphotericin antibiotic pastille/paste, and sucralfate) [4], management of acute pain, particularly that associated with oral mucositis, remains with the use of intravenous analgesics, particularly when oral medications are no longer effective or tolerated.

The association between hematological response to immunosuppression and pain remains poorly described. This observational study sought to quantify the relationship between white cell count and pain score. The impact of analgesic use on pain score was characterized. The temporal relationship between white cell count and pain score was described using a delayed effect model characterized by an equilibration half‐time.

## 2 Methods

This was a prospective, observational study conducted at Starship Children's Hospital, Auckland, New Zealand, from January 2021 through to December 2023. Patients between 6 months and 16 years of age suffering mucositis who were admitted to the pediatric oncology and hematology ward were eligible for inclusion. Initial routine analgesia comprised 6 hourly intravenous (IV) tramadol (1–2 mg/kg) and acetaminophen 15 mg/kg. Children were referred to the Acute Starship Pain Service for IV pain medication (opioids and ketamine) for further mucositis analgesic management. The parents and children were invited to participate in the study and consent was obtained from guardians. Assent was obtained from participants 5 years and above. Ethical approval for this study was given by the Auckland Health Research Ethics Committee, reference AH23434 with locality approval from Auckland District Health Board, reference A+9382.

### 2.1 Data Collection

The intravenous opioids (oxycodone and morphine) used by the participants in this study were administered by ambulatory infusion pumps (CAAD, Smiths Medical MD Inc., St Paul Minnesota, USA) using intermittent boluses and continuous infusions. The children were assessed by the Pediatric Pain Service as to the suitability of the child for Nurse Controlled Analgesia (NCA) or Patient Controlled Analgesia (PCA). Nurse Controlled Analgesia tended to be for the younger children younger than 6 years, with the older children opting for PCA. In the case of co‐analgesia, ketamine was delivered as an infusion (0.6–2.4 mg/kg/h) with no bolus option.

Analgesic opioid infusion devices allowed nursing staff to increase the bolus dose up to 20 μg/kg with a maximum background rate of 10 μg/kg/h on a patient using a PCA, and a maximum background infusion of 30 μg/kg/h on a patient prescribed a NCA. The PCA had a 5‐min lockout and the NCA a 15‐min lockout for a bolus dose. The 24‐h total use of IV opioids was recorded along with the bolus and background infusion and number of days the participant used the IV modalities. Morphine was the dominant intravenous opioid used for analgesia. Oxycodone was used in some children who had renal impairment or adverse effects with morphine. Oxycodone dose was converted to morphine equivalents (conversion factor, 1:1) for analysis.

All participants in this study had daily blood samples obtained from their central access catheters at 0600 h; these blood profiles included a full blood count and electrolytes. Hematology measures were analyzed using the Roche XN‐2000‐BB Hematology Analyzer (Sysmex America Inc., Lincolnshire, IL 60069, U.S.A). The lowest WCC reported was 0.1 × 10^9^/L, while that for neutrophils was 0.01 × 10^9^/L [5]. If the participant was neutropenic with no WCC recorded, this WCC was reported as zero.

The white cell counts, neutrophil count, 24‐h pain scores and oral mucositis score [6] was recorded every morning. Pain score was taken on the morning pain round with the numerical score from zero to 10 used. The FLACC score (facial expression; leg movement; activity; cry; and consolability) was used in younger children or those participants who were unable to verbalize their pain scores [7]. Daily Oral mucositis scores were taken using the Oral Assessment Guide which scores oral health (8–24); 8 being normal and 24 being severe mucositis [8].

### 2.2 Modeling

#### 2.2.1 Models

A two‐compartment model was used to describe pain relief. There was a temporal delay that related observed White Cell Count (WCC) and pain score. The compartment for observed WCC was related to an effect compartment (WCCe) by an equilibration rate constant (Keq). This rate constant was expressed as an equilibration half‐time (Teq) (Equation 1)

(1) $$\mathrm{Teq}=\frac{\mathrm{Log}\left(2\right)}{\mathrm{Keq}}$$

The relationship between the WCC and pain score (EFFECT_PAIN_) was characterized using a sigmoid fractional E_MAX_ model (Equation 2).

(2) $${\text{EFFECT}}_{\text{PAIN}}=\frac{{E_{\mathrm{MAX}}}_{\text{Pain}}\times {\text{WCCe}}^{\text{HILLe}}}{{\text{WCCe}}^{\text{HILLe}}+{{\mathrm{ED}}_{50\;\mathrm{WCC}}}^{\text{HILLe}}}$$

where the maximum pain score decrease ($E_{{\mathrm{MAX}}_{\text{Pain}}}$) is related to the total WCC and the WCC concentration at which a 50% decrease in pain occurs (ED_50 WCC_) in the effect compartment. HILLe is the Hill exponent describing the slope parameter for WCC effect [9].

The impact of opioids (expressed as morphine equivalents, EFFECT_MORPH_) on pain score was described using Equation (3).

(3) $${\text{EFFECT}}_{\text{MORPH}}=\frac{{E_{\mathrm{MAX}}}_{\text{MORPH}}\times {\text{MORPH}}^{\text{HILLm}}}{{\text{MORPH}}^{\text{HILLm}}+{{\mathrm{ED}}_{50\;\text{MORPH}}}^{\text{HILLm}}}$$

where MORPH is the morphine dose expressed as μg/kg/h. E_MAX MORPH_ is the maximum effect attributable to morphine. The ED_50 MORPH_ is the morphine dose (μg/kg/h) at which a 50% decrease in pain occurs. HILLm is the Hill exponent describing the slope parameter for morphine effect.

The impact of ketamine (EFFECT_KET_) on pain score was described as using Equation (4).

(4) $${\text{EFFECT}}_{\mathrm{KET}}=\frac{{E_{\mathrm{MAX}}}_{\mathrm{KET}}\times {\mathrm{KET}}^{\text{HILLk}}}{{\mathrm{KET}}^{\text{HILLk}}+{{\mathrm{ED}}_{50\;\mathrm{KET}}}^{\text{HILLk}}}$$

where KET is the ketamine dose expressed as mg/kg/h. E_MAX KET_ is the maximum effect attributable to ketamine The ED_50 KET_ is the ketamine dose (mg/kg/h) at which a 50% decrease in pain occurs. HILLk is the Hill exponent describing the slope parameter for ketamine effect.

The observed patient pain score was predicted using all three models describing effect (Equation 5).

(5) $$\begin{array}{c} \text{PAIN SCORE}=E_{0}\times \left(1-{\text{EFFECT}}_{\text{PAIN}}\right)\\ \;\times \left(1-{\text{EFFECT}}_{\text{MORPH}}\right)\times \left(1-{\text{EFFECT}}_{\mathrm{KET}}\right) \end{array}$$

The baseline pain (*E*_0_) is that pain score described by children administered oral acetaminophen and tramadol. The model is described in Supporting Information (Supporting Information 1. Mucositis NM‐TRAN Control Stream).

#### 2.2.2 Computation

Population parameter estimates were obtained using nonlinear mixed effects models (NONMEM 7.5.1 ICON Development Solutions, USA) with first‐order conditional estimation and a convergence criterion set to three significant digits. Population parameter variability (PPV) was described using an exponential model for the random effect variables (*η*); these variables were assumed to have a mean of zero and variance denoted by *ω* ^2^ (Equation 6).

(6) $$P_{i}=P_{\mathrm{TV}}e^{\mathrm{\eta i}}$$

where *P* is the parameter (e.g., E_MAX_, ED_50_) for the *i*th individual, *P*_*TV*_ is the typical value for that parameter and *η* is the random effects variable. Residual unidentified variability (RUV) was modeled using both proportional and additive residual error models.

#### 2.2.3 Model Selection

A decrease in the value of the objective function (OFV) provided by NONMEM indicated an improvement to the model. The likelihood ratio test with alpha = 0.01 was used to assess a significant improvement in the fit. However, this assessment is mostly applicable to nested models and was only applicable to certain parts of modeling in this current analysis (e.g., addition of an equilibration rate constant). A visual predictive check (VPC) for the PKPD model was generated based on simulations of model predictions including random effects. Biological plausibility of parameter estimates and inspection of VPC plots served as a guide during model building. Bootstrap methods were used to evaluate parameter uncertainty [10]. A total of 100 bootstrap replications were used to estimate parameter averages and confidence intervals (CI). Results from the population models are presented as parameter estimates, together with their 90% CI. Population parameter variability is expressed as an apparent coefficient of variation obtained from the square root of the variance estimate.

## 3 Results

**FIGURE 1:**
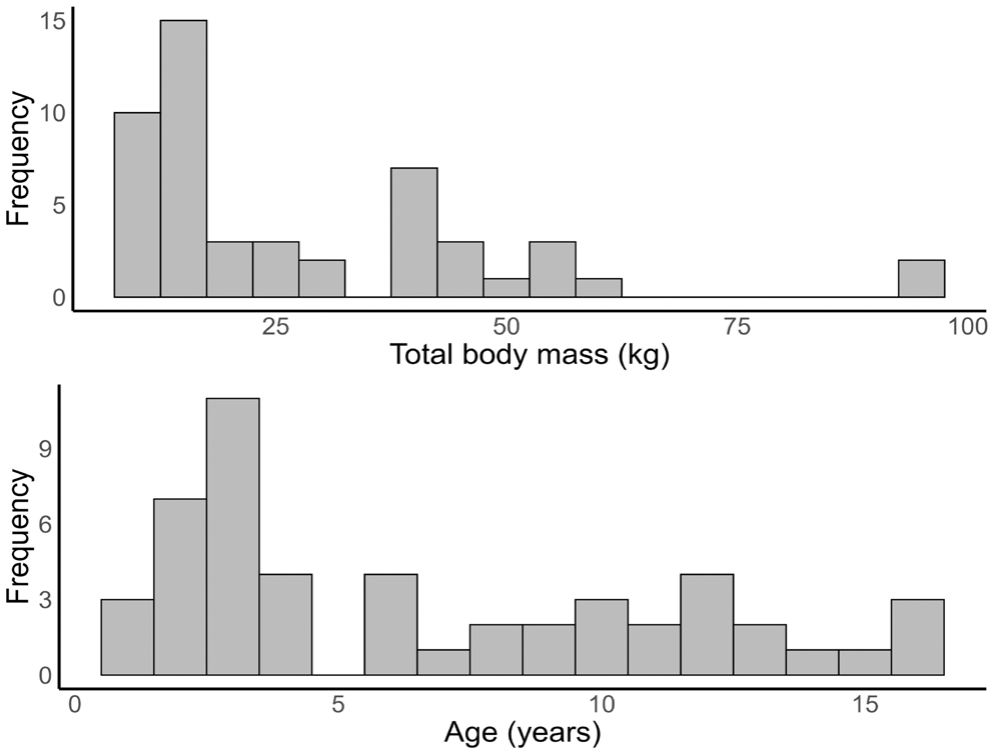
Distributions of total body mass (kg) and age (years) for children included in the study.

**FIGURE 2:**
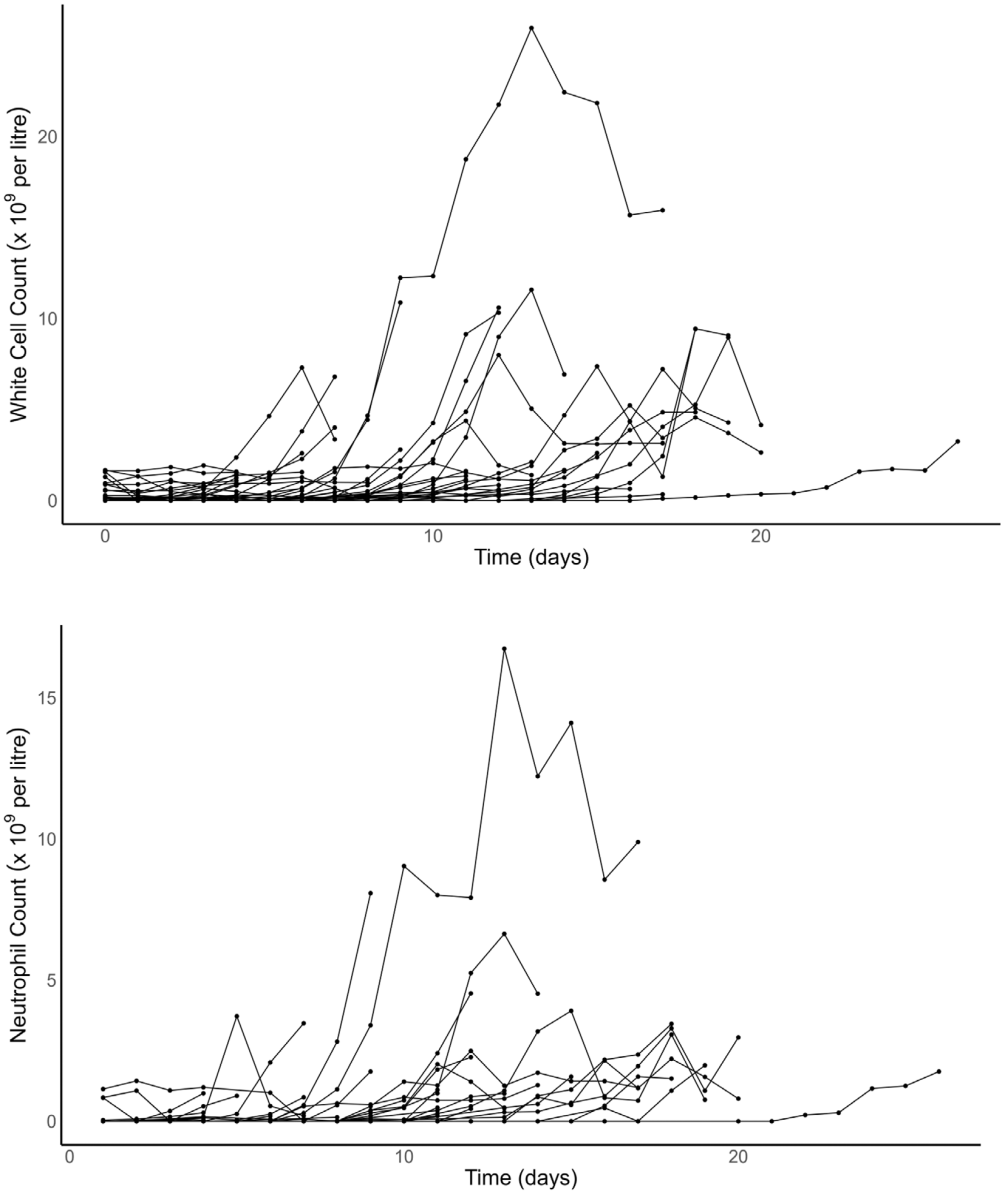
The white cell count (normal range 4–11 × 10^9^/L) changes over time is shown in the upper panel. The neutrophil count (normal range 1.9–7.5 × 10^9^/L) changes over the study time is shown in the lower panel. There is reasonable correlation between both white cell and neutrophil counts.

There were 50 patients, 19 females and 31 males who used pain modalities for mucositis pain. The median age was 4 years and 2 months (range 6 months to 16 years). Age and weight distributions are shown in Figure 1. Children suffered mixed pathology and 30 of the 50 children had a bone marrow transplant (Table 1). The neutrophil count was unrecordable in many children suffering from mucositis. Both the WCC and the neutrophil count increased at a mean of 9 days (Figure 2). These cell changes generally paralleled each other up until 2 weeks (*R* ^2^ = 0.86). Pain scores after 2 weeks were compromised by concomitant pathology (e.g., rectal fistulae, viral respiratory infection, hemorrhagic cystitis, and abdominal surgery). Mucositis scores before 2 weeks (13.2 SD 6.4) were similar in those who continued to require analgesia after 2 weeks (12.6 SD 4.7).

**TABLE 1:** Diagnostic categories for children suffering mucositis.

Diagnosis	Number	Bone marrow transplant
Neuroblastoma	12	10
Acute lymphoblastic leukemia	11	6
Acute myeloid leukemia	4	4
Burkitt lymphoma	4	0
Posterior fossa tumor	4	0
Medulloblastoma	2	1
Diamond‐Blackfan anemia	2	1
Myelomonocytic leukemia	2	2
Wilms tumor (Nephroblastoma)	1	0
Hepatoblastoma	1	1
Hunter Syndrome (Mucopolysaccharidosis type II)	1	1
Kostmann Syndrome (Severe congenital neutropenia)	1	1
Peripheral T‐cell lymphoma (Non‐Hodgkin Lymphoma)	1	0
Hemophagocytic lymphohistiocytosis	1	1
Non‐germinomatous germ cell tumor	1	1
Post‐transplant lymphoproliferative disorder	1	0
Mixed‐phenotype acute leukemia	1	1

**FIGURE 3:**
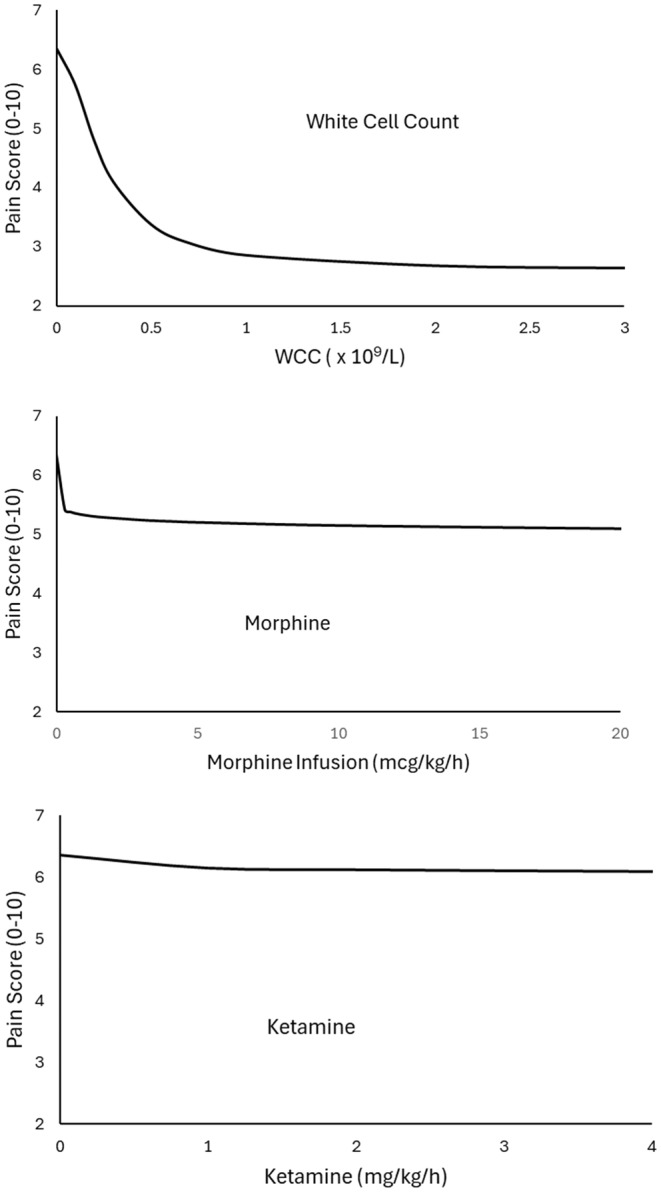
The relationships between WCC, morphine, ketamine, and pain score are shown graphically. The white cells have considerable impact on observed daily pain scores. The addition of morphine to children already given paracetamol, nonsteroidal anti‐inflammatories, and tramadol had little impact. Ketamine had even less effect that morphine.

**FIGURE 4:**
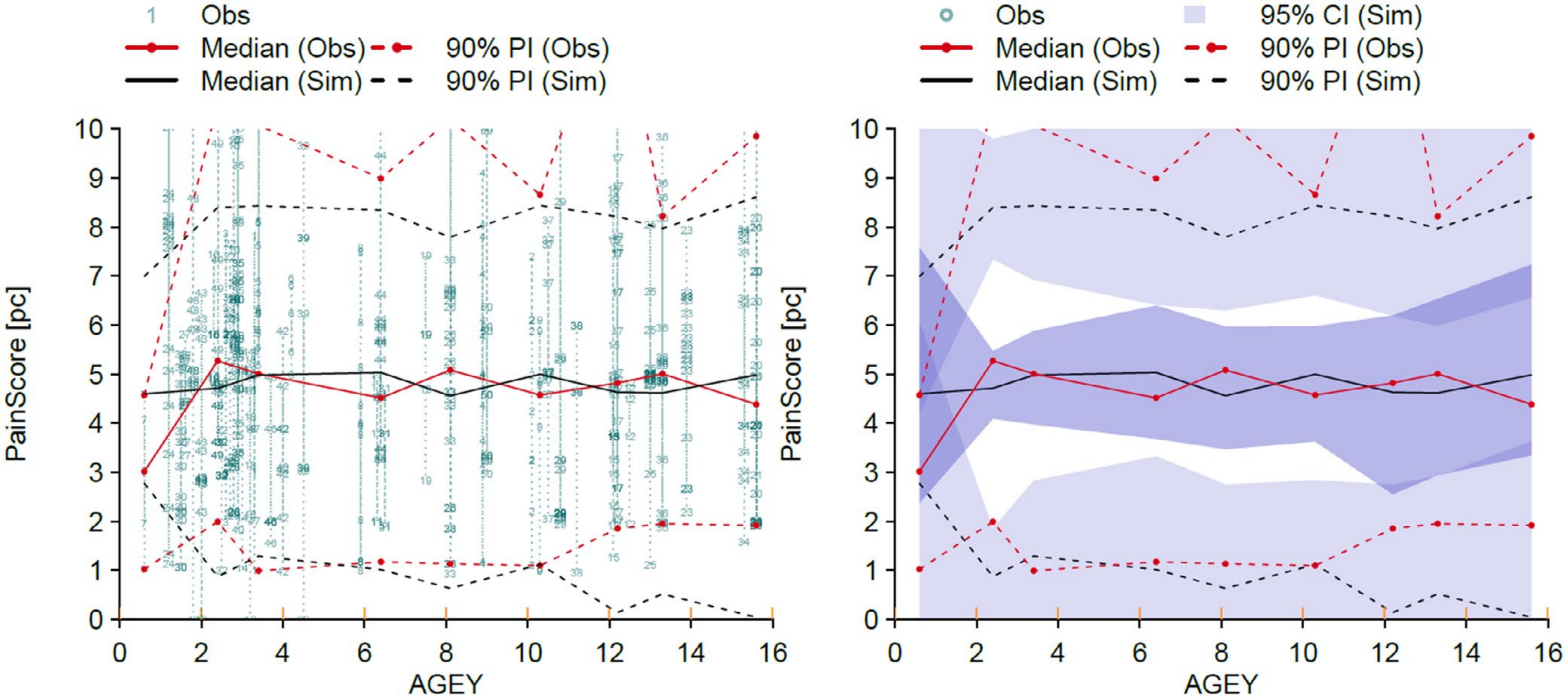
Mean pain score changes with age. There was considerable within patient and between patient variability over the study period, but mean pain score was 4.5/10 and this was consistent across all ages. Observation percentiles from the observed data are given in black (median, solid line; 10th and 90th percentile, dashed line). These are overlaid with model predictions (median, 10th and 90th prediction percentiles given in red; solid and dashed lines, respectively). Shaded areas are 95% confidence intervals for the prediction percentiles.

We were unable to establish a relationship between pain and mucositis score. Consequently, mucositis score was not further investigated as a covariate. The estimated relationships between WCC, morphine and ketamine and pain score are shown graphically in Figure 3. The temporal delay between the rise in WCC and pain response was 0.29 days. The initial pain score (*E*_0_, estimated in those children already started on treatment with paracetamol and tramadol) was 6.3 (maximum pain 10). The maximum pain reduction was 59% of that initial pain score. Morphine and ketamine further reduced pain; the maximum response for morphine was a 38% reduction and that for ketamine was 11%. There was considerable within‐patient and between‐patient variability over the study period, but mean pain score was 4.5/10 and this consistent across all ages (Figure 4). Parameter estimates for the model are shown in Table 2.

**TABLE 2:** Population parameter estimates for the WCC analgesic model. Parameter estimates and population variability displayed as medians determined from 100 bootstrap estimates. Residual unidentified variability: RUV; population parameter variability: PPV% = √variance.

Parameter	Estimate	PPV	90% CI
*E*_0_ (pain units (0–10)	6.4	20	6.08, 6.81
E_MAX PAIN_ (fractional pain units)	0.58	314	0.02, 0.97
ED_50 WCC_ × 10^9^/L	0.25	—	0.12, 0.63
HILLe	1.84	—	1.1, 8.9
Teq (h)	0.29	—	0.26, 0.40
E_MAX MORPHINE_ (fractional pain units)	0.38	—	0.29, 0.47
ED_50 MORPHINE_ (μg/kg/h)	8.3	—	1.3, 20
HILLm	0.13	—	0.1, 0.66
E_MAX KETAMINE_ (fractional pain units)	0.11	—	0.02, 0.18
ED_50 KETAMINE_ (mg/kg/h)	1.5	—	0.4, 1.9
HILLk	0.26	—	0.1, 0.36
RUV_ADD_ (pain units)	1.94	17	1.76, 2.14

*Note*: Parameters used to describe the E_MAX_ model: *E*_0_ is the baseline pain score described by children administered oral acetaminophen and tramadol at study initiation, E_MAX_ is the maximum pain score decrease attributable to WCC, morphine, and ketamine. The ED_50_ describes a 50% decrease in pain. The Hill coefficient relates to the steepness of the dose–response curve, Teq is the equilibration half‐time.

**FIGURE 5:**
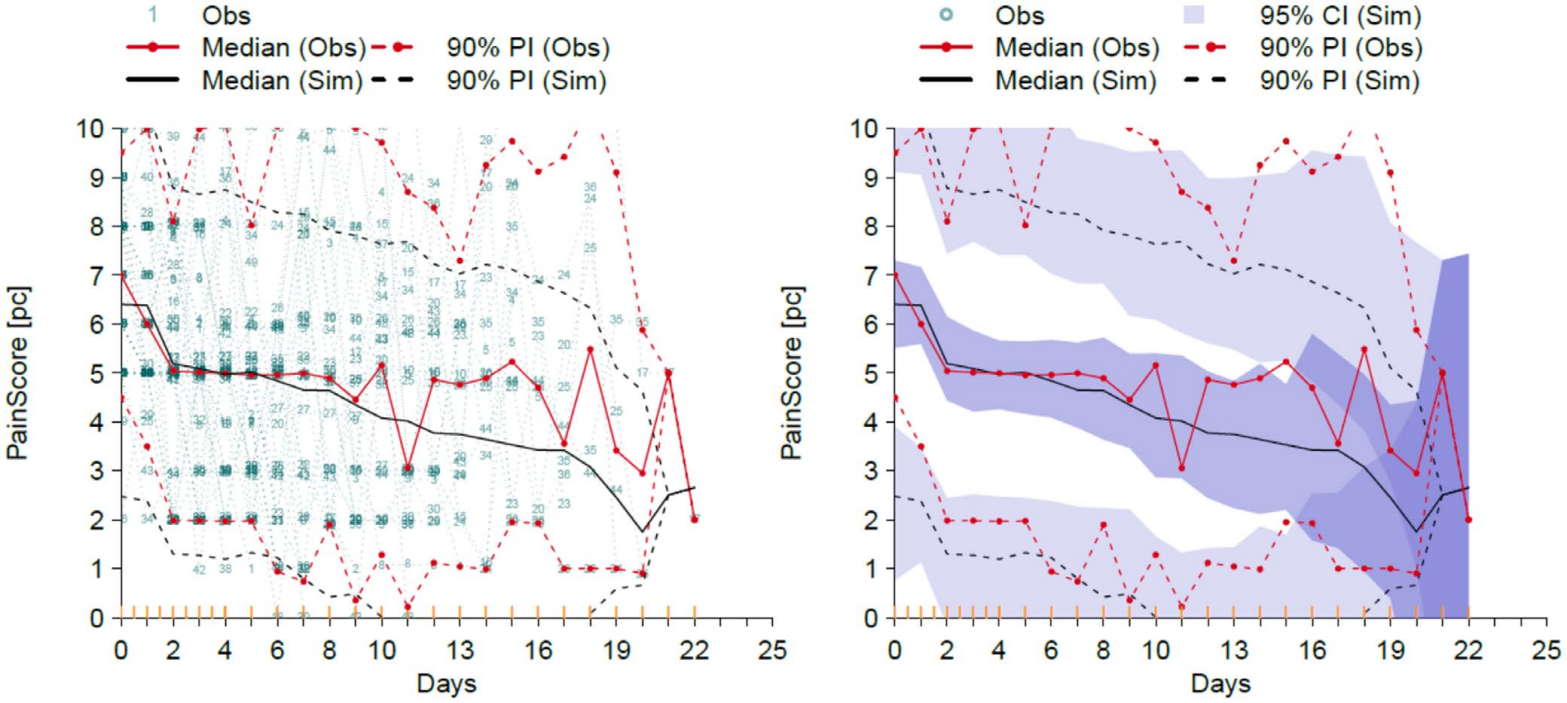
Visual predictive checks for the pharmacodynamic model. Observation percentiles from the observed data are given in black (median, solid line; 10th and 90th percentile, dashed line). These are overlaid with model predictions (median, 10th and 90th prediction percentiles given in red; solid and dashed lines, respectively). Shaded areas are 95% confidence intervals for the prediction percentiles. The observed and predicted percentiles are closely overlaid, with observation percentiles captured within the prediction confidence intervals, suggesting good model fit for the first 12 days.

The visual predictive check (VPC) [11] for pain scores over the 3‐week study period are shown in Figure 5. These plots demonstrate how well model predictions are consistent with observed pain scores. There is a reasonable correlation between model prediction and observed pain scores for the first 12 days only.

## 4 Discussion

Pain relief from mucositis is related to an increase in white cell count after a period of severe neutropenia, where white cell count is a surrogate for neutrophil count. This improvement in analgesia comes on early in the WCC recovery phase and can be described with a WCC that confers a 50% reduction in maximum pain, a WCC of 0.25 × 10^9^/L; essentially, as soon as neutrophils can be observed in routine blood testing. There is a delay in analgesic response of approximately 1 day (three to five times the equilibration half‐time of 0.29 days). This analgesic response to WCC changes (E_MAX PAIN_ 0.59) had greater dominance than analgesia achieved using either opioids (E_MAX MORPH_ 0.38) or ketamine (E_MAX KET_ 0.11). The mathematical model used to describe analgesia was reliable for the first 12 days of intravenous analgesic therapy only. Children with pain beyond this duration invariably had other pathology that contributed to pain; the source of pain was not only mucositis [2].

We have used models (mathematical equations) to describe our observations. Models used for this purpose can prove useful for clinical medicine because they quantify exposure–response relationships (e.g., response to morphine), provide clarity and insight (e.g., pain resolution related to WCC or reason for continued pain after WCC recovery), and provide scientific rationale for dose selection (the impact of higher doses of ketamine will be unhelpful). They can also be used to generate hypotheses and be used for theory enrichment [12].

A common issue with informing analgesic responses in children is understanding the time course of pain resolution and the impact of analgesics over and above that pain resolution [13]; disentangling physiological pain improvement from drug effects. One method is to determine placebo effect, a method with considerable ethical constraints [14]. Population modeling has been used to interpolate placebo effects from available and historical data [15], but such data are uncommon. Another modeling approach is to use a disease progression model that describes the underlying time course of a pathological process (e.g., Parkinson's disease [16], depression [17]). We have attempted to describe the resolution of pain using a disease progression model where the WCC is instrumental for pain resolution. The impact of morphine and ketamine could then be assessed relative to this baseline of pain resolution. The value of modeling and its translational impact on analgesia has been described [12, 18]. Modeling of pain data remains relatively uncommon, despite encouragement of this technique in children [19].

Symptoms of pain are reported as worse on day 8 after hematopoietic stem cell transplant [20]. The mean time from the start of chemotherapy to the onset of oral mucositis was 8.4 days (SD 4.0) with a median duration of mucositis of 7.0 days (range 4.0 to 10.5) in pediatric oncology patients undergoing cancer therapy [3]. The duration of mucositis was similar in this current study. Initial pain severity (*E*_0_ 6.4) was less than that described by others [2] because children were already managed with acetaminophen and tramadol before referral to the Acute Pain Service and inclusion in the study.

The impact of morphine and ketamine on analgesia remains poorly described. There was considerable within‐patient and between‐patient variability over the study period (Figure 4) and pain relief was directed at comfort rather than a target analgesic effect. We have attempted to use an E_MAX_ model to describe this analgesic response, but parameter estimates are imprecise, partly because the pain scores are imprecise with high variability. There are very few morphine pharmacodynamic models available for the quantification of acute pain [21, 22]. We anticipated a greater benefit from both morphine and ketamine. However, the poor response may, in part, be because children were already gaining benefit from other analgesics on trial entry. In addition, the analgesic assessment tool used does not include assessment around emotive and altered sensorium elements, both of which are influenced by opioids and ketamine.

## Ethics Statement

Auckland Health Research Ethics Committee, AH23434.

## Conflicts of Interest

Brian Anderson sits on the Editorial Board of the journal Pediatric Anesthesia. The authors have no other conflicts of interest to declare.

## Supporting information


Data S1.

## Data Availability

Data will be made available on reasonable request.
