# Supplementary material for: Mucositis Pain and Its Temporal Relationship to White Cell Count
Source: Paediatr Anaesth. 2025 Jan 6;35(4):302–9. doi: 10.1111/pan.15063 (PMC11883507; doi:10.1111/pan.15063)
Supplement: Supplementary file 1 — Data S1. [file PAN-35-302-s001.docx]

# Supplementary Material

## Mucositis NM-TRAN Control Stream

$DATA ..\MucosititsPKPD_WCCPain_MorphkgKet.csv

$ESTIM MAXEVAL=9999 NSIG=4 SIGL=12 NOABORT METHOD=CONDITIONAL INTERACTION

$SUB ADVAN13 TOL=9

$THETA

;WHITE CELL PD

(3,6.35, ) ;POP_E0_PAIN (BASELINE PAIN SCORE)

(0.01,0.591,1);POP_EMAX_PAIN (MAXIMUM EFFECT ON PAIN SCORE FROM WCC CHANGE)

(0.1,0.248,) ;POP_C50_WCC (WCC AT HALF-MAXIMAL PAIN EFFECT)

(1,1.83,10) ;POP_HILL (HILL EXPONENT FOR WCC PAIN RELATIONSHIP)

(0.2,0.287, 5) ;POP_TEQ (EFFECT COMPARTMENT HALF-LIFE IN DAYS)

;MORPHINE PD

(0.001,0.2,0.5) ;POP_ED50_MORPH (MORPHINE DOSE AT HALF-MAXIMAL EFFECT))

(0.001,0.373,1) ;POP_EMAX_MORPH (MAXIMUM EFFECT OF MORPHINE ON PAIN)

(0.1,0.103,7) ;POP_HILL_MORPH

; KETAMINE PD

(0.001,1.5,5) ;POP_ED50_KET (MORPHINE DOSE AT HALF-MAXIMAL EFFECT))

(0.001,0.108,1) ;POP_EMAX_KET (MAXIMUM EFFECT OF KETAMINE ON PAIN)

(0.1,0.29,7) ;POP_HILL_KET

;RESIDUAL UNIDENTIFIED VARIABILITY ON PAIN SCORE

(0,0.00017,) ;RUV_CVFX

(0,1.94,) ;RUV_SDFX

$OMEGA BLOCK(2)

0.0402 ;PPV_E0_PAIN

-0.746 58.4 ;PPV_EMAX_PAIN

$OMEGA

0 FIX ;PPV_C50_WCC

0 FIX ;PPV_HILL

0 FIX ;PPV_TEQ

0.0312 ;PPV_RUVFX

$SIGMA

1. FIX ;EPS1

$MODEL

COMP (WCC) ;WHITE CELL COUNT

COMP (WCCE) ;EFFECT COMPARTMENT WHITE CELL COUNT

COMP (PAIN) ;PAINSCORE

COMP (MORPH) ;MORPHINE EQUIVALENT MG/KG

COMP (KET) ;KETAMINE ML/H

$PK

E0_PAIN=POP_E0_PAIN*EXP(PPV_E0_PAIN)

LGTEMX_PAIN=LOG(POP_EMAX_PAIN/(1-POP_EMAX_PAIN))+PPV_EMAX_PAIN

EMAX_PAIN=1/(1+EXP(-LGTEMX_PAIN))

C50_WCC=POP_C50_WCC*EXP(PPV_C50_WCC)

HILL=POP_HILL*EXP(PPV_HILL)

TEQ=POP_TEQ*EXP(PPV_TEQ)

LGTEMX_MORPH=LOG(POP_EMAX_MORPH/(1-POP_EMAX_MORPH))

EMAX_MORPH=1/(1+EXP(-LGTEMX_MORPH))

ED50_MORPH=POP_ED50_MORPH

HILL_MORPH=POP_HILL_MORPH

LGTEMX_KET=LOG(POP_EMAX_KET/(1-POP_EMAX_KET))

EMAX_KET=1/(1+EXP(-LGTEMX_KET))

ED50_KET=POP_ED50_KET

HILL_KET=POP_HILL_KET

LN2=LOG(2)

KEQ=LN2/TEQ

$DES

DWCC=A(1)/POP_V1

DWCCE=A(2)/POP_V2

DPAIN=A(3)

DMORPH=A(4)

DKET=A(5)

DADT(1)=DWCC

DADT(2)=KEQ*(DWCC-DWCCE)

DADT(3)=DPAIN

DADT(4)=DMORPH

DADT(5)=DKET

$ERROR

WCCE=A(2)

PAIN=A(3)

MORPH=A(4)

KET=A(5)

;WHITE CELL COUNT

WCC50H=C50_WCC**HILL

WCCECH=WCCE**HILL

;MORPHINE

MORPH_HILL=MORPH**HILL_MORPH

ED50MH=ED50_MORPH**HILL_MORPH

;KETAMINE

KET_HILL=KET**HILL_KET

ED50KH=ED50_KET**HILL_KET

"IF (WCCE.LE.0) WCCE=1D-10

"IF (MORPH.LE.0) MORPH=1D-10

"IF (KET.LE.0) KET=1D-10

; CALCULATE EFFECT SIGMOIDAL RESPONSE CURVE

EFFECT_PAIN=(EMAX_PAIN*WCCECH)/(WCCECH+WCC50H)

EFFECT_MORPH=(EMAX_MORPH*MORPH_HILL)/(MORPH_HILL+ED50MH)

EFFECT_KET=(EMAX_KET*KET_HILL)/(KET_HILL+ED50KH)

;COMBINE WCC AND MORPHINE EFFECTS ON PAIN

PAIN=E0_PAIN*(1-EFFECT_PAIN )* (1 - EFFECT_MORPH)* (1 - EFFECT_KET)

IF (DVID.EQ.3) THEN

PROPFX=PAIN*RUV_CVFX

ADDFX=RUV_SDFX

SDFX=SQRT(PROPFX*PROPFX + ADDFX*ADDFX)*EXP(PPV_RUVFX)

Y=PAIN+SDFX*EPS1

ENDIF

$TABLE ID TIME ID E0_PAIN C50_WCC Y DV DVID WCCE
